# Preoperative serum levels of insulin-like growth factor-binding protein 2 predict prognosis of gastric cancer patients

**DOI:** 10.18632/oncotarget.14202

**Published:** 2016-12-26

**Authors:** Hoon Hur, Eun Ji Yu, In-Hye Ham, Hye-Jin Jin, Dakeun Lee

**Affiliations:** ^1^ Department of Surgery, Ajou University School of Medicine, Suwon, Republic of Korea; ^2^ Brain Korea 21 Plus Research Center for Biomedical Sciences, Ajou University, Suwon, Republic of Korea; ^3^ Department of Pathology, Ajou University School of Medicine, Suwon, Republic of Korea

**Keywords:** gastric cancer, tumor marker, insulin-like growth factor-binding protein 2, prognosis

## Abstract

It has been reported that serum insulin-like growth factor-binding protein 2 (IGFBP2) levels are elevated in various types of cancers. However, the clinicopathologic and prognostic implications of circulating IGFBP2 have never been investigated in gastric cancer. We tested IGFBP2 levels in the sera of 118 gastric cancer patients and 34 healthy controls using enzyme-linked immunosorbent assay (ELISA). The mean serum IGFBP2 level was significantly elevated in the gastric cancer patients compared to controls (805.23 ± 590.56 ng/ml vs. 459.61 ± 277.01 ng/ml; *P* < 0.001). Serum IGFBP2 levels were significantly higher in larger (> 6 cm) tumors (956.8 ± 734.0 ng/ml vs. 548.6 ± 364.0 ng/ml; *P* = 0.007) and in higher (T3/4) T stages (854.8 ± 621.4 ng/ml vs. 546.5 ± 315.1 ng/ml; *P* = 0.037). Multivariate Cox analysis showed that higher serum IGFBP2 level (> 400.01 ng/ml) was an independent prognostic factor predicting worse overall survival in patients with gastric cancer (hazard ratio (HR): 3.749, *P* = 0.034). When we divided patients into four groups based on blood IGFBP2 levels, survival was stratified. The HRs for death in the 3rd and 4th quartiles of serum IGFBP2 levels in comparison to that in the 1st quartile were 2.527 (*P* = 0.043) and 3.092 (*P* = 0.012). In conclusion, circulating IGFBP2 has potential as a biomarker predicting prognosis for gastric cancer patients.

## INTRODUCTION

Insulin-like growth factors (IGFs) are regulatory peptides with a number of biological functions, such as cell proliferation, differentiation, and anti-apoptosis [1, 2]. The IGF system consists of the two peptide ligands (IGF-I and IGF-II), six high-affinity IGF-binding proteins (IGFBP1 to IGFBP6), and two IGF receptors (IGF-IR and IGF-IIR) [3]. The action of IGFs are modulated by the IGFBPs in a positive or negative way, depending on tissue type and physiologic status [4]. In the circulation, over 95% of IGF-I and IGF-II are bound to the six IGFBPs [4]. In humans, IGFBP3 is the most abundant major IGFBP followed by IGFBP2 in the blood.

Unlike IGFBP3, which induces antitumor activity in different types of cancers [5, 6], IGFBP2 promotes tumorigenesis [7], cancer cell invasion [8], metastasis [9], and cancer stem cell expansion [10]. Previous reports demonstrated increased expression of IGFBP2 in various types of cancer tissue, including glioma [11], colorectal cancer [12], lung cancer [13], and gastric cancer [14, 15], and high expression of IGFBP2 was associated with worse survival. Since IGFBP2 is a secretory protein, it has been also observed that serum IGFBP2 was elevated in cancer patients compared to healthy individuals for ovarian cancer [16], colorectal cancer [17], and lung cancer [18]. High circulating IGFBP2 level was considered a poor prognostic factor in these tumors. Furthermore, serum IGFBP2 levels were correlated with tumor size in lung cancer [18], and the levels significantly dropped after curative resection in patients with colorectal cancer [17]; both observations implicate serum IGFBP2 as an indicator for tumor burden.

Although a few previous studies suggested the prognostic role of tissue IGFBP2 in gastric cancers [14, 15], the clinicopathologic and prognostic implications of circulating IGFBP2 have never been investigated in gastric cancer. Only a recent study demonstrated that serum IGFBP2 level was elevated in gastric cancer patients (2.2-fold change) compared to age- and sex-matched healthy controls, using a quantitative proteomic approach [19]. Herein, we investigated the diagnostic and prognostic role of circulating IGFBP2 in gastric cancer patients.

## RESULTS

### Clinicopathologic characteristics of patients

The clinicopathologic characteristics of the patients are summarized in Table 1. There were 118 gastric cancer patients (85 males and 33 females) with a median age of 61 years (range 27~90 years). The majority of the tumors were located in the lower third of the stomach (60 cases, 50.8%), followed by the mid-third (30 cases, 25.4%) and the upper third (28 cases, 23.7%). Histologically, diffuse-type of cancer was more prevalent (55 cases, 46.6%) than the intestinal type (47 cases, 39.8%) and mixed type (16 cases, 13.6%). Most of the cases were advanced gastric cancer (113 cases, 95.8%), and 78% of cases presented with regional lymph node metastasis. Distant metastasis was identified in 7 cases (5.9%) at the time of surgery.

**Table 1:** Demographic and clinical characteristics of the gastric cancer patients

Characteristics at surgery	No. of patients (%)
Age in years, median (range)	61 (27~90)
Gender	
male	85 (72)
female	33 (28)
Tumor size, median (range)	6 cm (1.2~22 cm)
Tumor location	
upper	28 (28.7)
mid	30 (25.4)
lower	60 (50.8)
Histology	
well differentiated	7 (5.9)
moderately differentiated	30 (25.4)
poorly differentiated	45 (38.1)
signet ring cell	31 (26.3)
mucinous	5 (4.2)
Lauren classification	
intestinal	47 (39.8)
diffuse	55 (46.6)
mixed	16 (13.6)
T stage	
T1	5 (4.2)
T2	14 (11.9)
T3	42 (35.6)
T4	57 (48.3)
N stage	
N0	26 (22)
N1	25 (21.2)
N2	14 (11.9)
N3	53 (44.9)
Lymphovascular invasion	
absent	38 (32.2)
present	80 (67.8)
Perineural invasion	
absent	58 (49.2)
present	60 (50.8)
M stage	
M0	111 (94.1)
M1	7 (5.9)

### Serum IGFBP2 levels in gastric cancer patients and healthy controls

**Figure 1:**
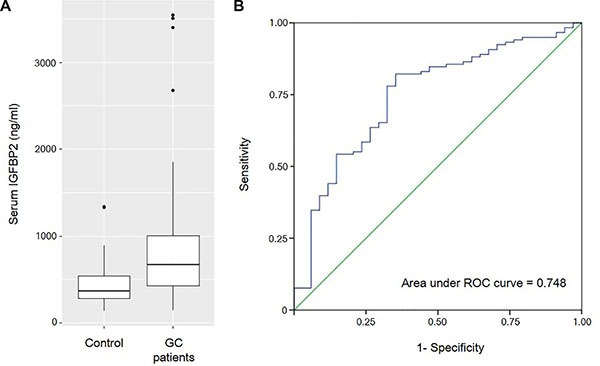
(**A**) The box plots show that the mean serum IGFBP2 level of gastric cancer patients are significantly higher than that of healthy controls (805.23 ± 590.56 ng/ml vs. 459.61 ± 277.01 ng/ml; *P* < 0.001). (**B**) Receiver operating characteristic (ROC) curve for the diagnosis of gastric cancer with serum IGFBP2 levels.

To test the diagnostic value of serum IGFBP2, we compared the circulating IGFBP2 levels between gastric cancer patients and healthy controls. The mean serum IGFBP2 level of gastric cancer patients was significantly higher than that of healthy controls (805.23 ± 590.56 ng/ml vs. 459.61 ± 277.01 ng/ml; *P* < 0.001) (Figure 1A). Meanwhile, three of the patients displayed extremely high levels of serum IGFBP2 (> 3000 ng/ml). Then, we generated a receiver operating characteristic (ROC) curve, which showed an area under curve of 0.748 (Figure 1B). At an optimal cut-off of 400.01 ng/ml, the sensitivity and specificity using serum IGFBP2 alone to differentiate the gastric cancer patients and healthy individuals were 79.7% and 64.7%, respectively. However, despite using the cut-off value of 400.01 ng/ml, McNemar's test showed that we cannot tell who has gastric cancer and who does not (*P* = 0.065).

### Correlation of serum IGFBP2 with clinicopathologic parameters

**Figure 2:**
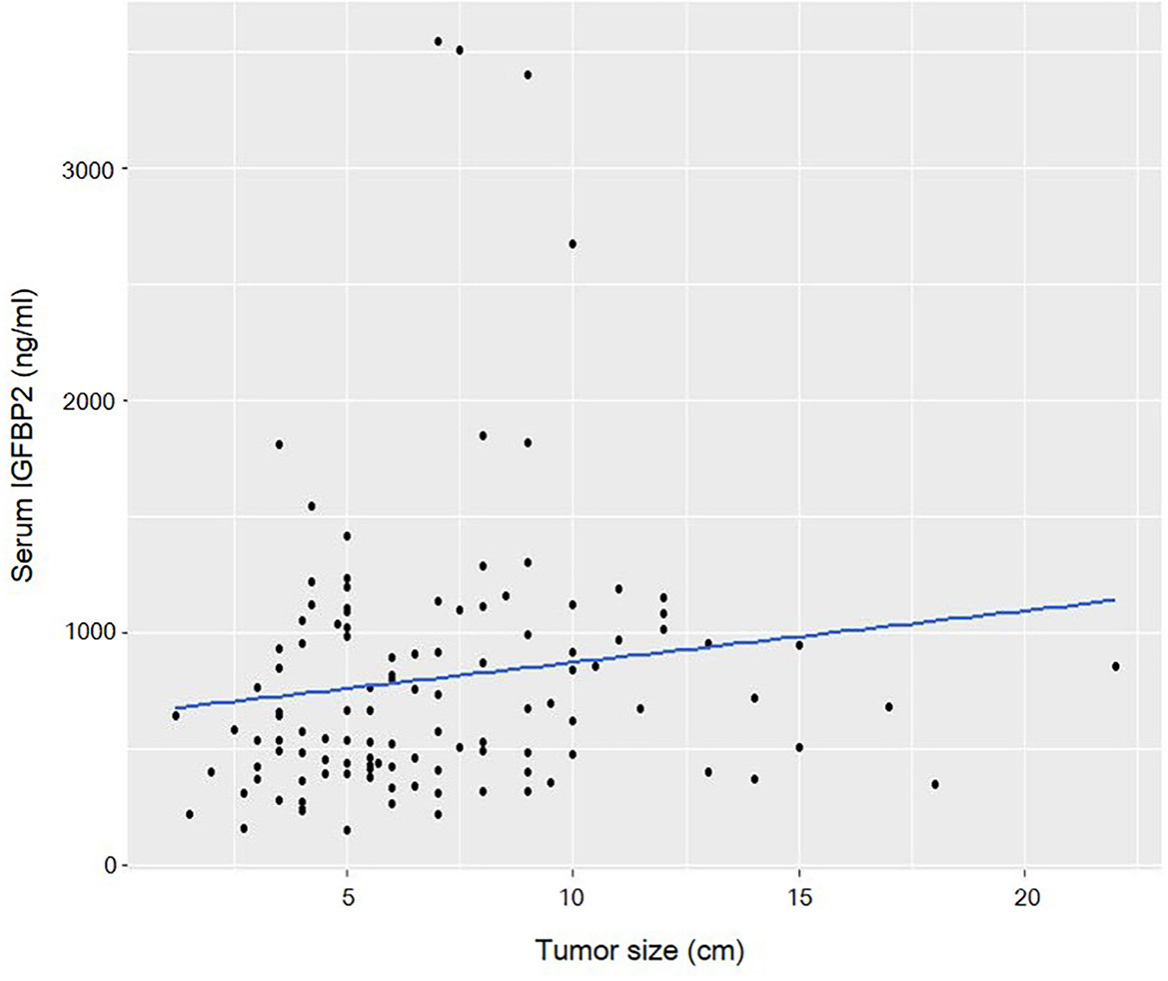
Scatter plot shows that circulating IGFBP2 levels are correlated with the tumor size (*R* = 0.241, *P* = 0.008)

**Figure 3:**
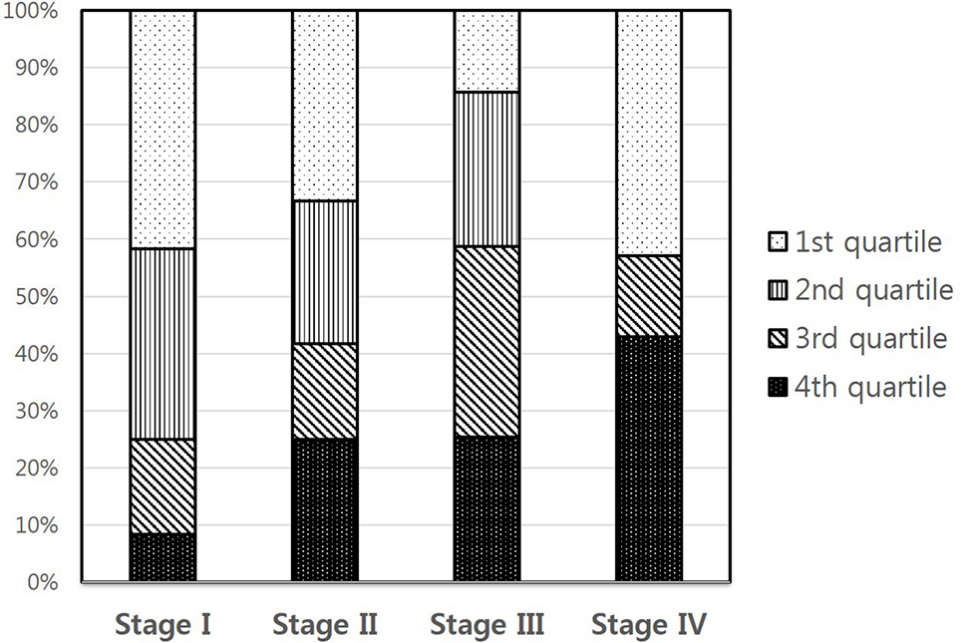
Correlation between IGFBP2 quartiles and TNM stages (*P* = 0.025)

Circulating IGFBP2 levels correlated well with the tumor size (*R* = 0.241, *P* = 0.008) (Figure 2), and IGFBP2 levels were significantly higher in larger tumors (> 6 cm) than those of smaller ones (956.8 ± 734.0 ng/ml vs. 548.6 ± 364.0 ng/ml; *P* = 0.007) (Table 2). Serum IGFBP2 levels in higher T stages (T3/4) were significantly higher than those in T1/2 stages (854.8 ± 621.4 ng/ml vs. 546.5 ± 315.1 ng/ml; *P* = 0.037). Patients with tumors having lymphovascular invasion also showed significantly higher serum IGFBP2 levels (*P* = 0.022). IGFBP2 levels were higher in higher (N2/3) N stage (878.6 ± 630.6 ng/ml vs. 708.7 ± 530.5 ng/ml; *P* = 0.124) and higher (III/IV) TNM stages (751.5 ± 603.5 vs. 543.0 ± 280.6 ng/ml; *P* = 0.109), but did not reach the statistical significance. The differences in IGFBP2 values according to age, gender, and histology were not significant. When we divided patients into four groups based on their blood IGFBP2 levels, we observed that patients with higher IGFBP2 quartiles at surgery were more likely to have advanced disease (Figure 3).

**Table 2:** Correlation of serum IGFBP2 levels with clinicopathological variables

Variables		*n*	IGFBP2 ng/ml (mean ± SD)	*P*-value
Age (years)	≤ 60	58	715.2 ± 518.3	0.105
	> 60	60	785.7 ± 520.3	
Gender	Male	85	785.7 ± 520.3	0.568
	Female	33	855.5 ± 756.1	
Tumor size (cm)	≤ 6	60	548.6 ± 364.0	0.007*
	> 6	58	956.8 ± 734.0	
Histology	differentiated	37	755.1 ± 422.7	0.538
	undifferentiated	81	828.1 ± 657.5	
Lauren classification	intestinal	47	859.8 ± 567.6	0.418
	diffuse+mixed	71	769.0 ± 610.6	
T stage	T1/2	19	546.5 ± 315.1	0.037*
	T3/4	99	854.8 ± 621.4	
N stage	N0/1	51	708.7 ± 530.5	0.124
	N2/3	67	878.6 ± 630.6	
LVI	absent	38	624.8 ± 550.5	0.022*
	present	80	890.9 ± 596.5	
PNI	absent	58	793.2 ± 511.0	0.831
	present	60	816.7 ± 667.0	
TNM stage	I/II	48	543.0 ± 280.6	0.109
	III/IV	70	751.5 ± 603.5	

LVI, lymphovascular invasion; PNI, perineural invasion.

Differentiated includes well and moderately differentiated adenocarcinomas. Undifferentiated includes poorly differentiated, signet ring cell, and mucinous adenocarcinomas.

*Significant at *P* < 0.05.

### Association of serum IGFBP2 with clinical outcomes

**Figure 4:**
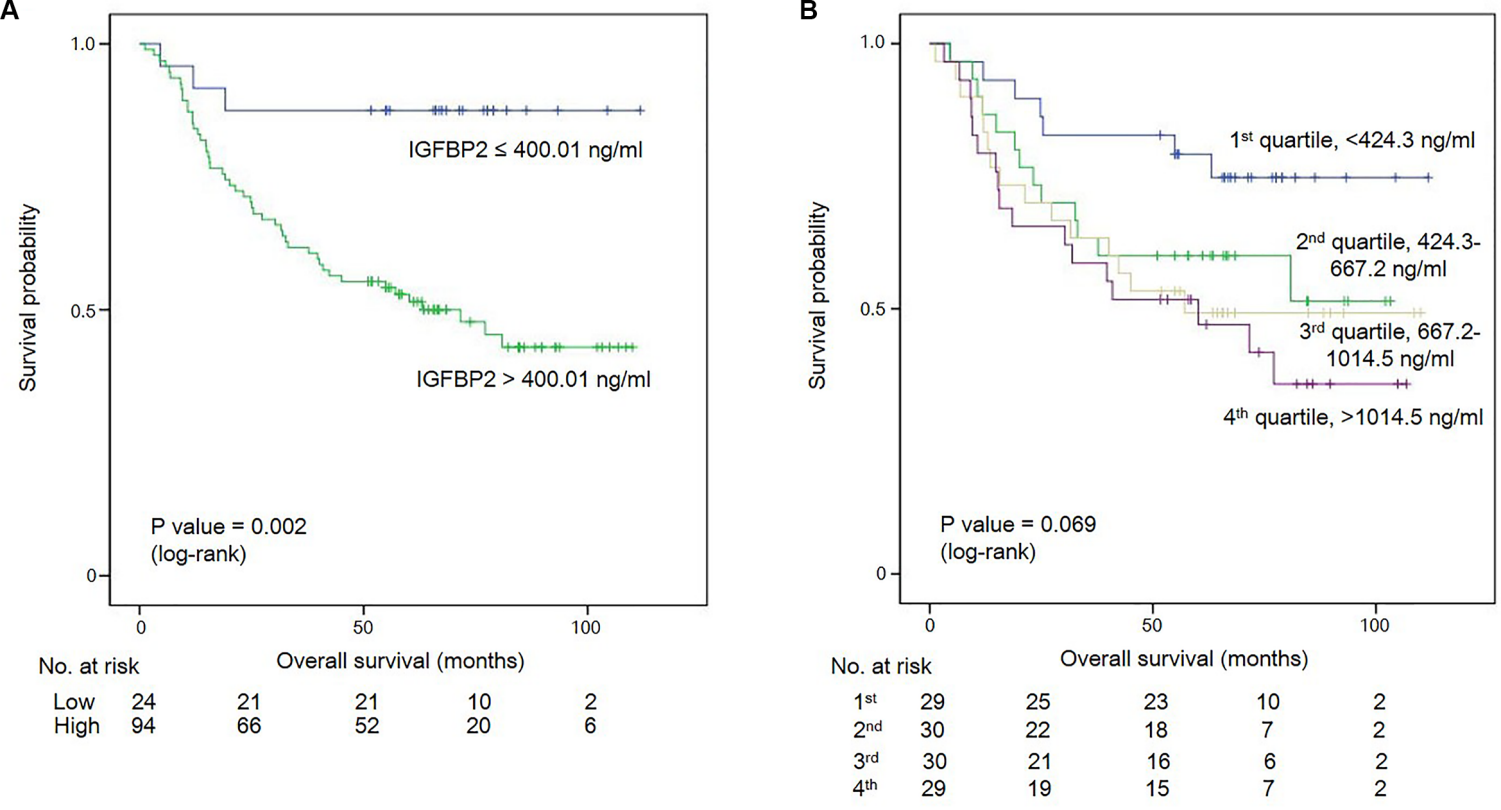
(**A**) Kaplan-Meier survival curves for overall survival at the serum IGFBP2 cut-off of 400.01 ng/ml. (**B**) Kaplan-Meier survival curves for gastric cancer patients subdivided into four groups based on their blood IGFBP2 levels.

With the cut-off of 400.01 ng/ml, patients with higher serum IGFBP2 levels (> 400.01 ng/ml) had a significantly lower 5-year overall survival rate (55.3% vs. 87.5%, *P* = 0.002) than patients with lower IGFBP2 values (Figure 4A). Univariate Cox analysis revealed that large tumor size (> 6 cm), undifferentiated histology, higher T stage (T3/4), higher N stage (N2/3), presence of lymphovascular invasion, and presence of perineural invasion along with high circulating IGFBP2 level (hazard ratio (HR): 5.221, *P* = 0.006) were significantly associated with worse overall survival (Table 3). Multivariate analysis showed that only higher N stage (N2/3) (HR: 2.858, *P* = 0.005) and higher serum IGFBP2 levels (HR: 3.749, *P* = 0.034) were independent prognostic factors predicting worse overall survival in patients with gastric cancer. When we divided patients into four groups based on their blood IGFBP2 levels, we found that IGFBP2 appeared to stratify survival, although as a whole, it did not reach the statistical significance (*P* = 0.069; Figure 4B). The mean survival time for the 1st, 2nd, 3rd, and 4th quartiles were 91.2, 68.0, 66.0, and 58.2 months, respectively. The HR for death in 3rd and 4th quartiles in comparison to that in the 1st quartile was 2.527 (*P* = 0.043) and 3.092 (*P* = 0.012), respectively (Table 4).

**Table 3:** Univariate and multivariate analyses of the overall survival rate according to the clinicopathologic variables

Variables		Univariate analysis			Multivariate analysis		
		HR	95% CI	*P*-value	HR	95% CI	*P*-value
Age	> 60 vs. ≤ 60 years	1.281	0.741–2.212	0.375	-	-	-
Gender	male vs. female	1.553	0.798–3.024	0.195	-	-	-
Tumor size	> 6 cm vs. ≤ 6 cm	1.899	1.088–3.312	0.024*	0.980	0.535–1.795	0.947
Histology	undifferentiated vs. differentiated	1.993	1.023–3.881	0.043*	1.849	0.941–3.633	0.075
Lauren classification	diffuse/mixed vs. intestinal	1.541	0.862–2.754	0.145	-	-	-
T stage	T3/4 vs. T1/2	4.356	1.351–14.042	0.014*	1.479	0.402–5.441	0.556
N stage	N2/3 vs. N0/1	3.883	2.022–7.458	< 0.001*	2.858	1.371–5.958	0.005*
LVI	present vs. absent	2.374	1.218–4.628	0.011*	1.197	0.590–2.432	0.618
PNI	present vs. absent	1.950	1.113–3.414	0.02*	1.420	0.793–2.542	0.238
IGFBP2	high vs. low	5.221	1.625–16.769	0.006*	3.749	1.107–12.695	0.034*

HR, hazard ratio; CI, confidential interval; LVI, lymphovascular invasion; PNI, perineural invasion.

*Significant at *P* < 0.05.

**Table 4:** Univariate survival analysis for serum IGFBP2 levels with the Cox regression model

IGFBP2	HR	95% CI	*P*
1st quartile	1		0.086
2nd quartile	2.068	0.824–5.186	0.122
3rd quartile	2.527	1.030–6.204	0.043*
4th quartile	3.092	1.281–7.464	0.012*

HR, hazard ratio; CI, confidential interval.

*Significant at *P* < 0.05.

## DISCUSSION

In the present study, we tested the serum IGFBP2 levels in healthy controls and gastric cancer patients. Serum IGFBP2 levels were significantly increased in patients with gastric cancer. In addition, for the first time, we evaluated the prognostic role of serum IGFBP2 in gastric cancer and demonstrated that high serum IGFBP2 level was an independent poor prognostic factor in patients with gastric cancer, although T stage, tumor size, or lymphovascular/perineural invasion were not independent prognostic factors. In addition, we showed that circulating IGFBP2 level can distinguish those patients with aggressive disease, which may be used for individualized management. Higher serum IGFBP2 levels were likely to be found in large tumors and in higher tumor stages, presenting circulating IGFBP2 level as a marker for tumor burden, as previously indicated [17, 18].

Elevated blood IGFBP2 level has been identified in various human malignancies such as ovarian cancer [16], prostate cancer [20], lung cancer [18], pancreatic cancer [21], glioma [22] and colon cancer [17]. These collective observations strongly suggest that IGFBP2 may be involved in the development and progression of malignant tumors in general. In the present study, although the mean serum IGFBP2 level in gastric cancer patients was increased about 2-fold than that of healthy controls, its diagnostic sensitivity and specificity were only modest (79.7% and 64.7%, respectively), and it could not differentiate the cancer patients from controls statistically even at the optimal cut-off. However, since the number of healthy controls was limited in number, this needs to be investigated in larger cohort studies. The observation of very high levels of serum IGFBP2 (> 1000 ng/ml) in several patients indicate a possible association between circulating IGFBP2 and certain benign conditions. This should be examined in the future. Meanwhile, blood IGFBP2 was reported to be inversely correlated with body mass index in healthy people [23]. In our study, since we used old serum samples, we cannot guarantee the quality of the individual samples, and this might be our drawback.

There are no specific tumor markers for gastric cancer. Although carcinoembryonic antigen (CEA) and carbohydrate antigen (CA) 19-9 are the most commonly used in clinical practice for gastric cancer, the sensitivity and specificity of these markers are generally low [24, 25]. In addition, these markers may be increased in benign conditions. Serum CEA can be increased in some smokers or patients on dialysis, and serum CA 19-9 can be elevated in cholecystitis, liver cirrhosis, and acute pancreatitis [26, 27]. Since serum IGFBP2 is correlated with tumor size, clinical stage, and prognosis with relatively high sensitivity and specificity, IGFBP2 may be a good candidate biomarker for gastric cancer patients. In particular, combining serum IGFBP2 with other serological markers or screening tools possibly increase its sensitivity and specificity. This warrants further investigation.

The physiologic roles of IGFBP2 in cancer are under active investigation. Previously, it was recognized that IGF-II/IGFBP2 complex may partly bind to the extracellular matrix (ECM) from where IGF-II may be liberated by proteolysis [28], suggesting the role of IGFBP2 as a storage pool for IGF-II in the tumor microenvironment [17]. Das et al. reported that secreted IGFBP2 interactes with αVβ3 integrin, activates the phosphoinositol-3-kinase (PI3K)/AKT pathway, then leads to the upregulation of the proangiogenic factor vascular endothelial growth factor (VEGF)-A and ultimately triggers angiogenesis in melanoma [29]. In addition, another study revealed that the PI3K/AKT pathway promoted the expression of IGFBP2 in MCF-7 breast cancer cells, and LY294002 (a PI3K inhibitor) directly reduced IGFBP2 production via the SP-1, which is a transcription factor involved in IGFBP2 [30]. Since IGFBP2 is a critical point in the crosstalk of several signaling pathways, IGFBP2 may become a candidate target of therapeutic potential. In fact, it was recently observed that the IGFBP2-neutralizing antibody leads to a reduction in the levels of phosphorylated EGFR, STAT3, and AKT in human glioma cells [31].

In conclusion, we demonstrate significantly elevated serological levels of IGFBP2 proportional to the tumor size and stage in gastric cancer patients, and show that high serum IGFBP2 level is an independent prognostic factor predicting poor survival in patients with gastric cancer. Therefore, circulating IGFBP2 may become a good candidate biomarker for gastric cancer patients. Large prospective studies are required to confirm the role of IGFBP2 as a promising tumor marker in patients with gastric cancer.

## MATERIALS AND METHODS

### Patients and samples

The experimental cohort consisted of 118 sera of the gastric cancer patients who underwent curative surgical resection with standard lymphadenectomy from March 2005 to November 2008 at Ajou University Hospital. The control group consisted of 34 (15 men and 19 women) healthy individuals whose esophagogastroduodenoscopy findings were normal, ranging in age from 25~49 years, Sera were collected at the Ajou Human Bio-Resource Bank before surgery and were frozen at −80°C until use. Patients who were pathologically diagnosed with stage II or higher stages of gastric cancer were recommended for treatment with adjuvant chemotherapeutic regimens including 5-fluorouracil. The clinicopathologic data were retrieved from the patients’ medical records. The median follow-up duration was 57.5 months. For statistical reasons, tumors were classified into differentiated type (well differentiated and moderately differentiated adenocarcinoma) and undifferentiated type (poorly differentiated adenocarcinoma, signet ring cell carcinoma, and mucinous adenocarcinoma). The pathological stages were adjusted based on the AJCC 7th edition [32]. This study was conducted in accordance with the ethics code of the World Medical Association (Declaration of Helsinki), and was approved by the Institutional Review Board (IRB) of Ajou University Hospital.

### Enzyme-linked immunosorbent assay (ELISA)

Serum IGFBP2 concentration were measured by an ELISA using IGFBP2 ELISA kit (Abcam, Cambridge, UK). Assays were performed following manufacturer's instruction, and the IGFBP2 levels were determined blindly without knowing any clinical information. Serum samples were diluted 1:200 with dilution buffer. Diluted samples (100 μl) and serially diluted IGFBP2 standards were added to each well of 96-well plates coated with IGFBP2 antibody, and then were incubated for 2.5 hours at room temperature with gentle shaking. After incubation, wells were washed with wash buffer and incubated with biotinylated anti-Human IGFBP2 antibody and horse radish peroxidase (HRP)-conjugated streptavidin. The enzymatic activity of HRP was determined using substrate tetramethylbenzidine (TMB) by measuring absorbance at 450 nm. All samples were examined in duplicate.

### Statistical analyses

Statistical analyses were performed using SPSS for Windows (version 22.0, SPSS Inc., Chicago, IL, USA). Values of serum IGFBP2 were expressed as mean ± standard deviation (SD). Circulating IGFBP2 levels between cases and controls were compared using the independent Student *t*-test, and the differences between cases and controls at the optimal cut-off was examined using the McNemar's test. Differences in serum IGFBP2 according to the demographic data were examined by an independent Student *t*-test or one-way ANOVA with post-hoc Tukey HSD analysis, as appropriate. Correlation between continuous variables were analysed using a Spearman rank test. IGFBP2 quartiles and TNM stages were compared using the Chi-square test by two-sided linear-by-linear association. Survival curve was constructed using the Kaplan-Meier method, and the difference was compared using a log-rank test. Univariate and multivariate analyses of survival were performed using a Cox proportional hazards regression model. For further analysis, gastric cancer patients were divided into four groups based on their blood IGFBP2 levels (1st, 2nd, 3rd, and 4th quartiles), and each quartile had 29 or 30 patients. A *P value* less than 0.05 was considered statistically significant. All the reported *P* values are two-sided. Some graphs were generated using the R statistical language v. 3.2.2. software.
